# Supplementary material for: Conditional chemoconnectomics (cCCTomics) as a strategy for efficient and conditional targeting of chemical transmission
Source: eLife. 2024 Apr 30;12:RP91927. doi: 10.7554/eLife.91927 (PMC11060718; doi:10.7554/eLife.91927)
Supplement: Figure 3—source data 3. [file elife-91927-fig3-data3.zip › Figure 3 source data3 part 2.zip!/Figure 3-Source data 3-part2/1053f2-1-CRISPResso_Report_y3hlioj1.zip!/CRISPResso_on_1053f2_4/1a.Read_barplot.pdf]

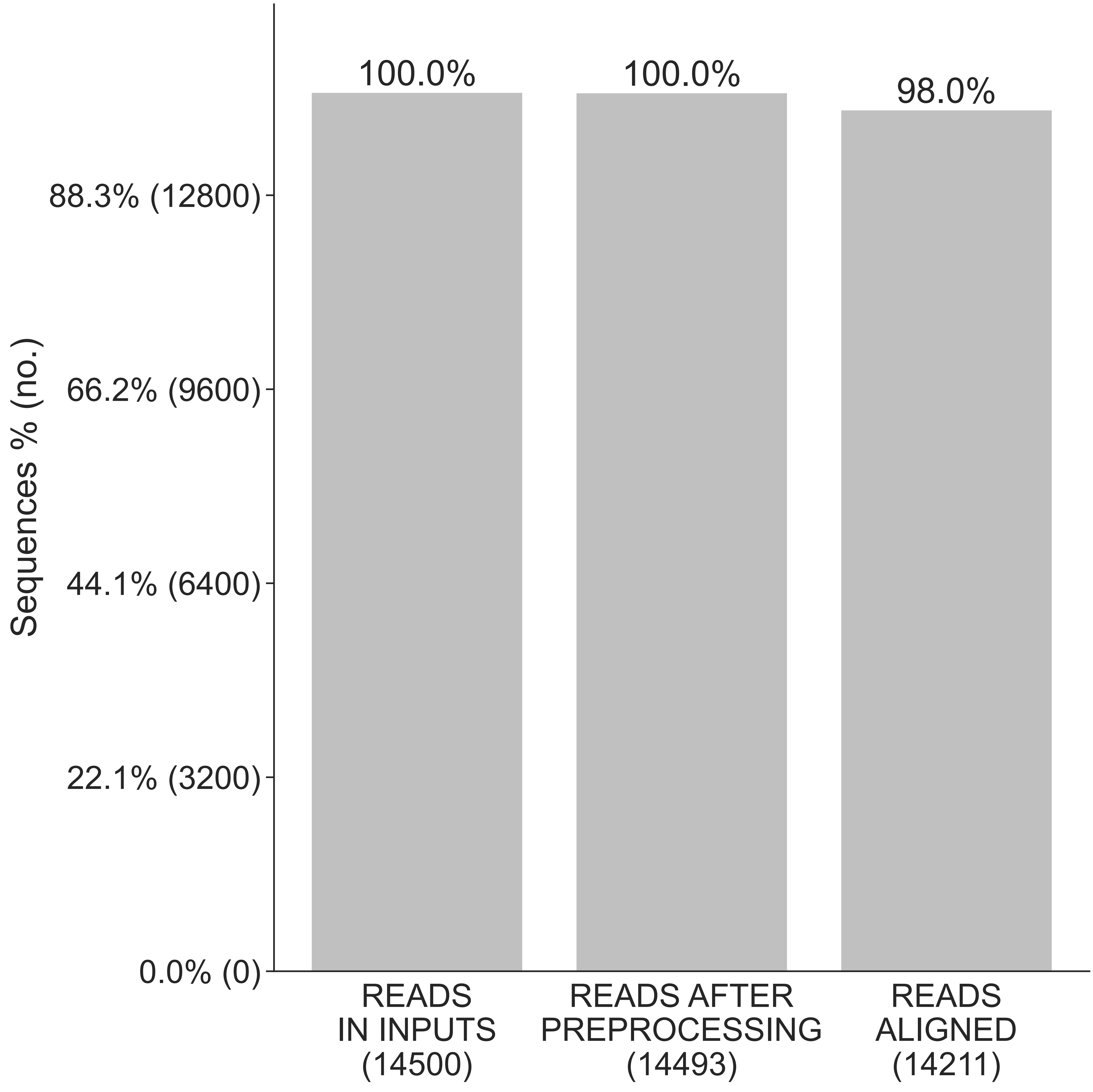
100.0%
100.0%
98.0%
Sequences % (no.)
88.3% (12800)
66.2% (9600)
44.1% (6400)
22.1% (3200)
0.0% (0)
READS IN INPUTS (14500)
READS AFTER PREPROCESSING (14493)
READS ALIGNED (14211)
